# Causal relationship between oral microbiome, circulating inflammatory proteins and lung cancer: A Mendelian randomization study

**DOI:** 10.1097/MD.0000000000051142

**Published:** 2026-10-16

**Authors:** Songhe Ping, Dan Li, Jian Sun, Kelong Tao, E. Qin, Renqi Tang, Hongguang Shan, Han Ye, Meng He

**Affiliations:** a School of Medicine, Shaoxing University, Shaoxing, Zhejiang, China; b Department of Hematology, Shaoxing People’s Hospital (The First Affiliated Hospital of Shaoxing University), Shaoxing, China; c Department of Respiratory and Critical Care Medicine, Shaoxing People’s Hospital (The First Affiliated Hospital of Shaoxing University), Shaoxing, China; d Department of Gastrointestinal Surgery, Shaoxing People’s Hospital (The First Affiliated Hospital of Shaoxing University), Shaoxing, China; e School of Medicine, Zhejiang University, Hangzhou, China.

**Keywords:** circulating inflammatory proteins, cystatin D, lung cancer subtypes, Mendelian randomization, oral microbiome

## Abstract

Recent research showed that oral microbiomes (OMs), as the main source of lung microbiomes, were associated with lung cancer (LC), but the causal relationship and mechanisms remain unclear. This study aimed to explore the immune mechanisms of LC through the “OM - inflammatory proteins - LC” axis. Instrumental variables covering 43 oral microbial taxa, 91 circulating inflammatory proteins, and LC subtypes were obtained from large-scale genome-wide association study summary data of European origin. We employed Mendelian randomization (MR) analysis to validate causal links between the OM and LC and to assess the mediation effects of inflammatory proteins. The primary statistical method was the inverse variance weighted method, supplemented by leave-one-out analysis, MR-Egger regression, and Cochran’s Q test to assess heterogeneity and pleiotropy. Finally, we calculated the effect size of inflammatory proteins as mediators. The results suggested that 9 oral microbial taxa and 20 inflammatory proteins were significantly associated with LC and its subtypes. Key findings included: Genus *Rothia* increased the risk of squamous cell carcinoma. At the species level, *Haemophilus parainfluenzae* protected against lung adenocarcinoma, whereas *Megasphaera micronuciformis* prevented small cell LC. Among the inflammatory proteins, the protein S100-A12 elevated adenocarcinoma risk, and STAM binding protein increased the risk of squamous cell carcinoma. Protective proteins included artemin (squamous cell carcinoma), cystatin D (adenocarcinoma), and interleukin 18 (both subtypes mentioned above). *M. micronuciformis* and overall LC had a causal relationship in which cystatin D played a mediating role. This study reveals a potential causal link between the OM, circulating inflammatory proteins, and LC. The protective effect of *M. micronuciformis* was mediated by cystatin D, highlighting a novel immune pathway. These findings provide biomarkers for early detection of LC and therapeutic targets for LC intervention.

## 1. Introduction

Lung cancer (LC) is one of the most common types of cancer worldwide today. With almost 2.5 million new cases, accounting for 12.4% of all cancers worldwide, LC was the most common cancer in 2022, according to global cancer statistics from the International Agency for Research on Cancer.^[1]^ Small cell lung cancer (SCLC) and non-small cell lung cancer (NSCLC) are the 2 major types of primary lung cancer (LC) derived from the epithelial cells of the bronchial mucosa. NSCLC, as the most common type (about 85%), is primarily composed of squamous cell lung carcinoma (LUSC) and lung adenocarcinoma (LUAD) pathological types, exhibiting certain genetic and cellular heterogeneity.^[2–4]^ This genetic heterogeneity is of great significance for the pathogenesis and treatment of NSCLC. In particular, activating mutations in the epidermal growth factor receptor (EGFR) gene represent the most well-characterized oncogenic drivers in NSCLC, especially in LUAD, and EGFR-tyrosine kinase inhibitors (TKIs) have become the cornerstone of targeted therapy for EGFR-mutant NSCLC. In addition to somatic EGFR mutations, EGFR single nucleotide polymorphisms (SNPs) have also been studied for their potential association with the susceptibility, treatment response, and clinical outcomes of NSCLC.^[5–7]^ These findings highlight the importance of genetic factors in shaping the biological behavior and treatment response of NSCLC. However, the molecular heterogeneity of LC is not limited to classic genetic alterations; other biological factors involved in the occurrence and development of LC still need to be further clarified. Moreover, SCLC is recognized as a highly aggressive neuroendocrine carcinoma with a 5-year overall survival rate of only approximately 7%.^[8,9]^ Because of its neuroendocrine differentiation, several circulating neuroendocrine markers, such as chromogranin A, pro-gastrin-releasing peptide, and neuron-specific enolase, have been investigated and used for clinical diagnosis and prognostic assessment of SCLC.^[10]^ Although in recent years, with advances in early detection and treatment, the mortality of LC has declined, it remains the leading cause of cancer-related death worldwide. Early diagnosis, prevention, and therapeutic targets for LC still need to be further explored urgently.

The oral microbiome (OM), the second-largest microbial community in the human body, contains over 700 distinct kinds of bacteria. Of these, the majority belong to 7 phyla, such as Actinomycetota and Bacillota.^[11]^ A growing number of studies suggest that changes in the highly diverse microbiome of the oral cavity, the anterior entrance to the respiratory tract, may be potentially relevant to LC. A higher risk of LC is linked to a decrease in the alpha diversity of OM, while beta diversity is unrelated to this risk.^[12,13]^ H. Dean Hosgood and colleagues discovered that a larger abundance of Spirochaetia and Bacteroidetes could reduce the risk of LC, while Bacilli class and Lactobacillales order were associated with a higher LC risk.^[12]^ In addition, Emily Vogtmann et al found that a one standard deviation increase in oral *Streptococcus* abundance was related to a 1.14-fold increase in LC risk. They also observed that the risk of LC was higher for those who discovered *Peptoniphilus* and lower for those who detected *Peptostreptococcus*, *Eubacterium yurii*, or *Aggregatibacter*.^[13]^ A recent clinical cohort study revealed that *Streptococcus* was enriched in both the oral cavity and lungs of patients with synchronous multiple primary LC, and this bacterium may inhibit the apoptosis of tumor cells, thereby promoting the progression of LC.^[14]^ Undoubtedly, OM plays a significant role in the development of LC by causing dysbiosis or direct inhalation that alters the pulmonary microenvironment and inflammatory and immune response.

Circulating inflammatory proteins also have a dual function in the pathogenesis of LC. Increasing evidence indicates that a broad range of inflammatory cytokines, chemokines, growth factors, and other immune-related proteins are involved in LC, rather than being restricted to a small number of individual inflammatory mediators.^[15]^ These inflammatory factors can participate in multiple aspects of LC development, including tumor cell proliferation or death, angiogenesis, immune regulation, invasion, and metastasis. For example, both CCL9 and IL-23, signaling molecules produced by epithelial cells, promote Myc-driven tumor growth and maintenance by remodeling the LC microenvironment,^[16]^ whereas elevated circulating CDCP1 has been associated with an increased risk of developing LC.^[17]^ Furthermore, IL-4 and IFN-γ have been reported to help LC cells escape being killed by the immune system by regulating the distribution and function of T cells in the tumor immune microenvironment.^[18–20]^ Importantly, these examples represent only a subset of the inflammatory mediators that have been investigated in LC, highlighting the complexity and heterogeneity of the inflammatory network involved in LC. Therefore, large-scale protein quantitative trait locus (pQTL), which link genetic variants to protein abundance, provide an important resource for investigating the potential causal effects of genetically predicted circulating inflammatory proteins using Mendelian randomization (MR).^[21]^

MR is a genetic epidemiological technique that uses genetic variants as instrumental variables (IVs) to distinguish correlation and causation between exposure and outcome variables.^[22,23]^ Its advantage is that it is less susceptible to other biasing factors such as the environment, as the IVs selected are genetic variants inherited at conception. In recent years, with the extensive research on MR, the causal relationship between the gut microbiota, the largest microbial community in the body, and lung diseases such as LC and asthma has been explored, and the pathogenesis of lung diseases has been better understood at the level of the “lung - gut” axis.^[24–26]^ However, the OM may have a more direct impact on the lungs than the gut microbiota, due to the tight intertwining of anatomical areas between the mouth and lungs.

Current research indicates that LC is independently associated with inflammatory proteins and the OM. However, most of the published findings focus on correlation analysis, while the causal relationships among them remain unclear. It is also uncertain if inflammatory proteins are implicated as intermediary mediators in the causal effect of OM on LC. In this study, we performed MR analyses to explore the causal effects among these 3 factors. By investigating the “oral - inflammatory protein - lung” axis, this provides new insights to further elucidate tumor immunity and pathogenesis of LC.

## 2. Materials and methods

### 2.1. Study design

As depicted in Figure 1, our MR study consisted of three major analytical components. Firstly, we explored the causal effects of 43 OM taxa on LC, followed by reverse MR analysis to assess the possibility of reverse causality. Secondly, we systematically evaluated the causal effects of 91 circulating inflammatory proteins on LC to identify candidate proteins that may serve as mediators. Thirdly, we examined the causal effects of OM on the candidate inflammatory proteins identified above, and subsequently performed 2-step mediation analysis for proteins that were causally associated with both OM and LC, thereby quantifying the mediating role of inflammatory proteins in the OM–LC causal pathway.

**Figure 1.**
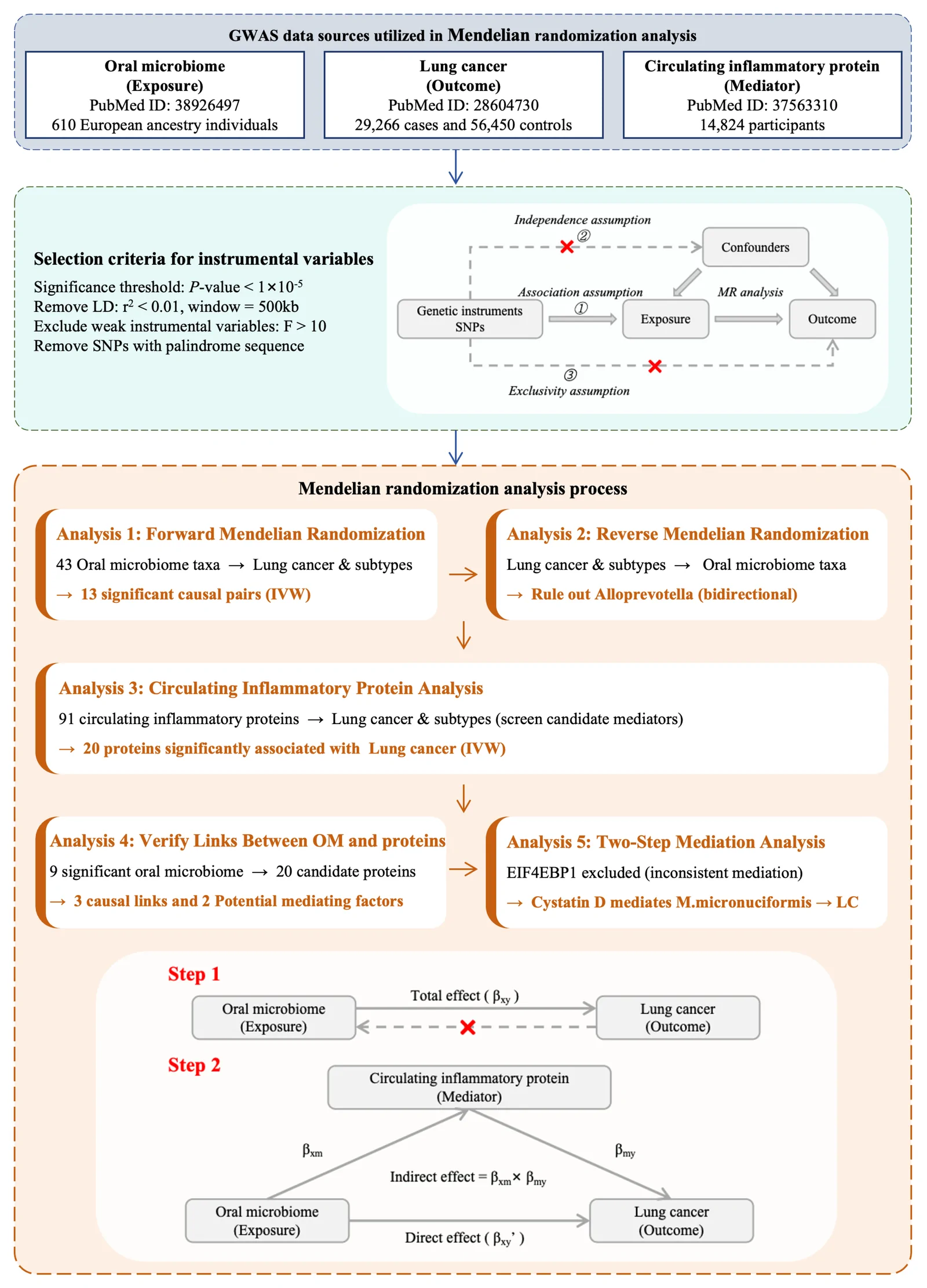
Flowchart. IVW = inverse variance weighted, LD = linkage disequilibrium, MR = Mendelian randomization, SNPs = single nucleotide polymorphisms.

Accordingly, OM taxa were considered exposure variables in the OM-to-LC and OM-to-inflammatory protein analyses, circulating inflammatory proteins were considered exposure variables in the inflammatory protein-to-LC analysis and potential mediators in the mediation analysis, and LC phenotypes were considered outcome variables. All of our analyses were performed in R software version 4.2.2 and used R packages such as TwoSampleMR. To reduce population stratification bias and to control for consistency in genetic structure, all Genome-wide association study (GWAS) data selected in our study were derived from European population pedigrees. In addition, these pooled data are all available for download and use in the GWAS Catalog (https://www.ebi.ac.uk/gwas/).

### 2.2. Data sources

#### 2.2.1. Oral microbiome

Table 1 summarized the sources of GWAS data. We obtained SNPs associated with human OM composition from a risk-stratified cohort study, which has been uploaded to the GWAS.^[27]^ In a cohort of 1657 participants, after filtering out first- and second-degree relatives, duplicate individuals, individuals identified as non-European ethnicity outliers, individuals with very high inbreeding coefficients, or individuals with incorrect gender labeling, 610 individuals passed the quality control criteria and provided saliva samples. These 610 saliva samples were subjected to 16S rRNA gene amplicon sequencing after DNA extraction. To reduce redundancy, bacterial characteristics were classified into 43 clusters based on similarity: 2 phyla, 1 class, 4 orders, 5 families, 12 genera, and 19 species.

**Table 1:** Details of GWAS data sources.

Phenotypes	GWAS ID	PMID	Journal	First author (yr)	Sample size	Ancestry
Oral microbiome	GCST90429799–GCST90429841	38,926,497	Sci rep	Stankevic E (2024)	610 participants	European
Circulating inflammatory proteins	GCST90274758–GCST90274848	37,563,310	Nat immunol	Zhao JH (2023)	14,824 participants	European
Lung cancer	GCST004744, GCST004746–GCST004750	28,604,730	Nat genet	McKay JD (2017)	29,266 cases and 56,450 controls	European

#### 2.2.2. Circulating inflammatory proteins

Summary-level GWAS data related to 91 circulating inflammatory proteins were derived from a genome-wide pQTL study analysis.^[28]^ The study recruited 11 cohorts containing 14,824 participants. In the original study, circulating inflammatory proteins were measured using the Olink Target-96 Inflammation immunoassay panel. The researchers initially measured 92 plasma proteins using the Olink Target-96 Inflammation immunoassay panel, but brain-derived neurotrophic factor was not included in the final study due to detection issues. The original pQTL study identified genetic variants associated with circulating levels of these proteins and provided summary-level genetic association statistics. In the present study, these 91 proteins were first treated as exposure traits in the “protein-to-LC” MR analysis. The subset of proteins showing evidence of association with LC or its histopathological subtypes was subsequently carried forward for the “OM-to-protein” analysis and considered as potential mediators in the “OM–LC” causal pathway.

#### 2.2.3. Lung cancer

Summary GWAS data for LC were obtained from a study by the International Lung Cancer Consortium.^[29]^ The study included 29,266 LC cases and 56,450 controls. Among them, based on different histopathological subtypes, the researchers classified LC patients into 11,273 LUAD, 7426 LUSC, and 2664 SCLC cases. In addition, based on the determination of smoking status, the study participants were categorized into 2 subgroups, including smokers (23,223 cases and 16,964 controls) and never-smokers (2355 cases and 7504 controls). These LC phenotypes were used as outcome traits in the corresponding MR analyses.

### 2.3. IVs screening

The reliability of the results of the MR analyses rests on three core assumptions: there is a reliable association between IVs and selected exposure factors (correlation assumption); IVs are independent of other confounders (independence assumption); and IVs affect outcomes only through exposure factors and not through other pathways (exclusivity assumption).^[22,23]^ Therefore, in assessing the impact of predictors on outcomes, we included SNPs as IVs and used the following screening criteria. Due to the limited sample size of OM, we uniformly adopted *P* < 1 × 10^−5^ as the significance threshold in order to obtain a larger number of SNPs. Second, genetic variants with similar genomic locations are more inclined to be inherited in nonrandom chains, and this co-inheritance phenomenon is called linkage disequilibrium (LD). To mitigate the effect of LD on the analysis results, we used a LD coefficient *r*^*2*^ of 0.01, a LD region width of 500 kb, and further excluded palindromic sequences and incompatible alleles. Weak IVs with *F* < 10 were excluded by calculating *F* statistic parameters.

### 2.4. MR analysis

To investigate the causal relationship between 43 OM taxa, 91 circulating inflammatory proteins, and LC and its pathological subtypes, 2-sample MR analyses were conducted. MR Egger, Weighted median, inverse variance weighted (IVW), Simple mode, and Weighted mode were used as the assessment methods in this study. Among them, IVW was our main analysis method. According to the requirement of two-sided statistical analysis, a *P* value < .05 was considered significant.

### 2.5. Sensitivity analysis

Heterogeneity between individual genetic variant estimates was further analyzed using IVW and MR-Egger methods. We calculated Cochrane’s *Q* statistic with its degrees of freedom and *P*-value. Cochrane’s *Q* statistic was used to measure the degree of heterogeneity among different genetic variants. Significant heterogeneity is indicated when *P < *.05, at which point we used a random-effects model instead of IVW as the primary research method.^[30,31]^

Although the MR Egger method itself corrects for horizontal pleiotropy to some extent, we further tested for the presence of horizontal pleiotropy by looking at the intercept term of the MR Egger regression. When *P < *.05, we considered that horizontal pleiotropy existed in the analyses. In other words, these SNPs may have an effect on the outcome through other pathways in addition to influencing the exposure factor, which violates the exclusivity assumption. Therefore, results with horizontal pleiotropy were not included in the subsequent analyses because of the high likelihood of bias in these results.

In addition, we used the “leave-one-out” analysis to test the sensitivity of MR results. We assessed whether a single SNP had a disproportionate effect on the overall estimate by leaving out 1 SNP at a time.

### 2.6. Reverse causality and mediation analysis

In exploring the causal relationship between OM and LC, we performed reverse MR analyses to rule out the possibility of a bidirectional association, which means changes in OM abundance from LC itself. To explore whether circulating inflammatory proteins play an intermediate mediator role between OM and LC, we conducted mediation analyses. Initially, we attempted to infer a more precise mediating effect using multivariate MR analysis, as this method allows for simultaneous consideration of both exposure and mediator effects on outcomes. However, due to sample size limitations and other issues, we finally used a 2-step MR approach to calculate the mediating effect.^[32–34]^ The total effect is β_xy_, the mediating effect is β_xm_ * β_my_, and the mediation ratio is (β_xm_ * β_my_)/ β_xy_.

## 3. Results

### 3.1. Identification of OM causally associated with LC

Based on the screening method described above, we finally obtained 2355 SNPs associated with the 43 OM taxa as IVs (Supplementary table S1, Supplemental Digital Content 1). Among these SNPs, the F statistic ranged from 19.78 to 36.62, with an average value of 21.88.

We explored the positive causal relationship between OM and LC using five analytical methods, including MR Egger et al When the results of these 5 methods met the *P*-values of one or more of them and were <.05, we preliminarily believed that there might be a potential causal relationship between the 2. Therefore, the results of the positive MR analyses showed that a total of 18 OM were potentially causally associated with overall LC or related subtypes (Figure 2B and Supplementary table S2, Supplemental Digital Content 2). These OM included four associated with LUAD, three associated with SCLC, two associated with LUSC, four associated with overall LC in never smokers, one associated with overall LC in smokers, and four associated with overall LC.

**Figure 2.**
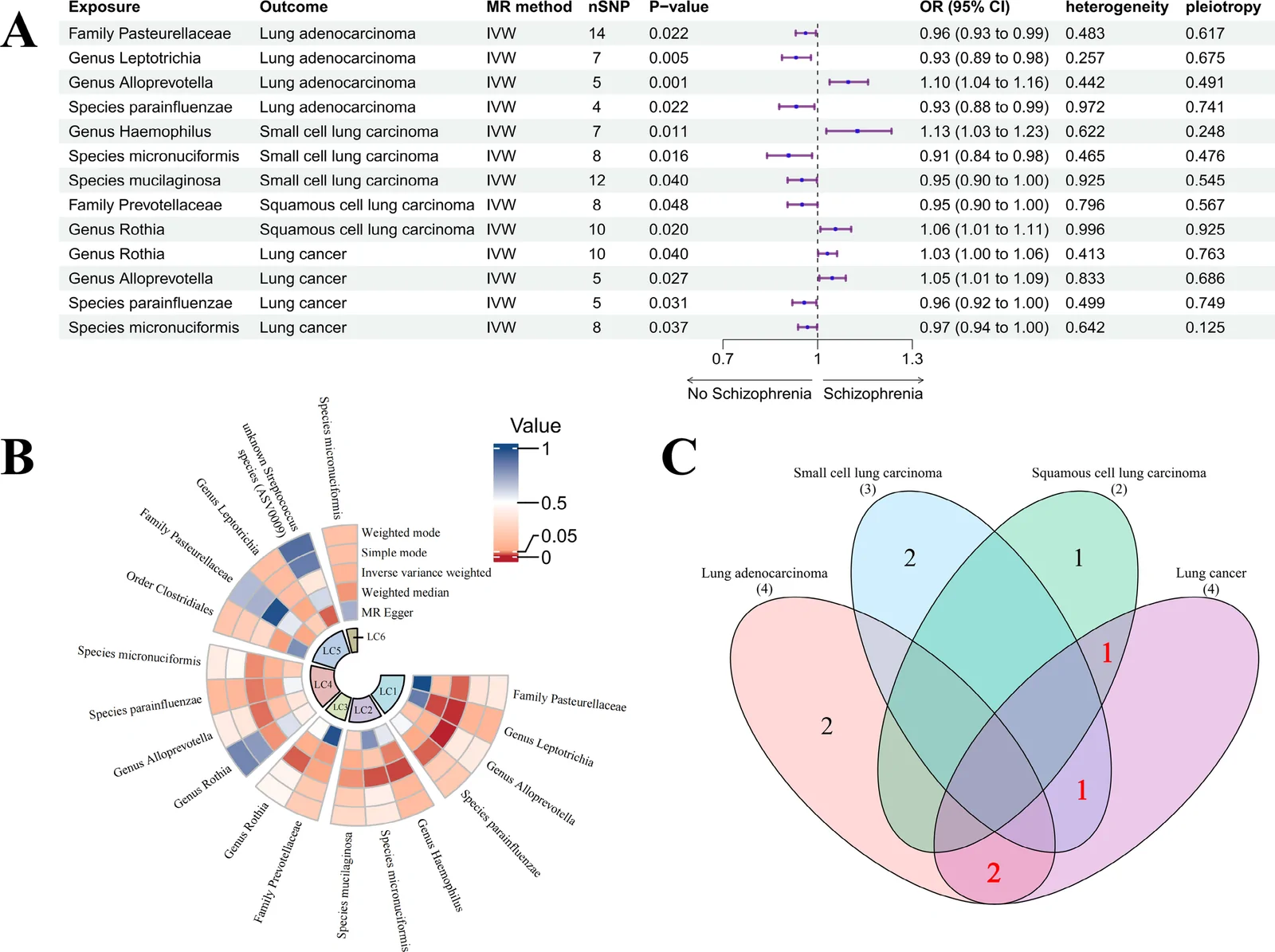
Results of positive MR analysis of oral microbiome on lung cancer. (A) Results of the IVW model and forest plot for positive MR analysis. (B) Circular heatmap of *P* values under 5 analysis methods. (C) Venn diagram showed the intersection of causality between different lung cancer subtype groups in the IVW model. IVW = inverse variance weighted, LC = Lung cancer, LC1 = lung adenocarcinoma, LC2 = small cell lung cancer, LC3 = squamous cell lung carcinoma, LC4 = overall lung cancer, LC5 = lung cancer in never smokers, LC6 = lung cancer in ever smokers, MR = Mendelian randomization; nSNP = number of single nucleotide polymorphisms.

The IVW model, as the main analysis method, has a high reference value and recognition of its results. IVW analysis results suggested that only 13 pairs of the above causal relationships were significant (Fig. 2A). Family Pasteurellaceae (OR = 0.96, 95%CI: 0.93–0.99), genus *Leptotrichia* (OR = 0.93, 95%CI: 0.89–0.98), and *Haemophilus parainfluenzae* (OR = 0.93, 95%CI: 0.88–0.99) were protective against LUAD. In contrast, genus *Alloprevotella* (OR = 1.10, 95%CI: 1.04–1.16) may raise the incidence of LUAD. *Megasphaera micronuciformis* (OR = 0.91, 95%CI: 0.84–0.98) and *Rothia mucilaginosa* (OR = 0.95, 95%CI: 0.90–1.00) decreased the probability of occurrence for SCLC, while the genus *Haemophilus* (OR = 1.13, 95%CI: 1.03–1.23) raised it. Furthermore, the risk of LUSC was decreased by family Prevotellaceae (OR = 0.95, 95%CI: 0.90–1.00), whereas it was increased by the genus *Rothia* (OR = 1.06, 95%CI: 1.01–1.11). More interestingly, the causal relationships, which indicated OM significantly associated with overall LC, also showed significance in MR analysis of the three LC pathological subtypes mentioned above (Fig. 2C), and they were directionally consistent. While genus *Rothia* (OR = 1.03, 95%CI: 1.00–1.06) and genus *Alloprevotella* (OR = 1.05, 95%CI: 1.01–1.09) could promote overall LC development, *H. parainfluenzae* (OR = 0.96, 95%CI: 0.92–1.00) and *M. micronuciformis* (OR = 0.97, 95%CI: 0.94–1.00), on the other hand, may inhibit LC. Moreover, neither smokers nor nonsmokers showed any appreciable significance in the IVW model for overall LC. All the names of the microbial groups in following figures were in the original format of the dataset and should correspond to the standard format in the text (ex. “Species parainfluenzae” refers to “*Haemophilus parainfluenzae*”).

The results of the heterogeneity test suggested that none of the above 13 causal relationships were significantly heterogeneous in the IVW model (Supplementary table S3, Supplemental Digital Content 3). The funnel plot (Fig. 3) demonstrated that the distribution of the included SNPs was roughly symmetric, suggesting that potential bias was less likely. The results of the MR-Egger analysis showed that the difference between the intercept term and 0 was not statistically significant (*P* > .05), meaning that there was no horizontal pleiotropy among those SNPs (Fig. 4 and Supplementary table S4, Supplemental Digital Content 4). After leave-one-out testing, we found that after excluding each SNP one by one, the results of MR analyses for the remaining SNPs were similar to those obtained using all SNPs (Fig. 5).

**Figure 3.**
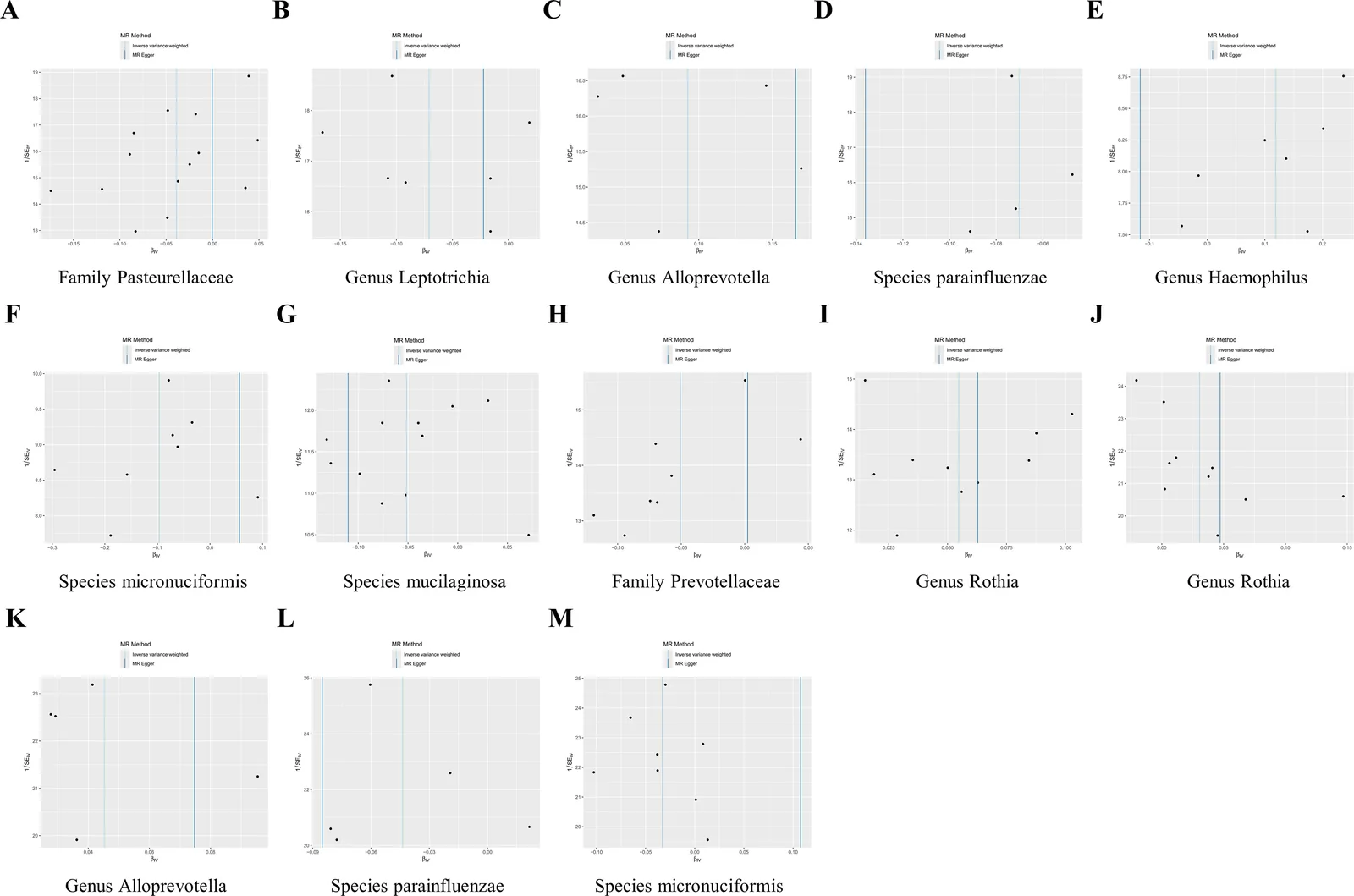
Funnel plot of 13 pairs of causal relationships of oral microbiome on lung cancer. (A) ~ (D) Causal relationships on lung adenocarcinoma: (A) family Pasteurellaceae, (B) genus Leptotrichia, (C) genus Alloprevotella, (D) Haemophilus parainfluenzae. (E)–(G) Causal relationships on small cell lung cancer: (E) genus Haemophilus, (F) Megasphaera micronuciformis, (G) Rothia mucilaginosa. (H)–(I) Causal relationships on squamous cell lung carcinoma: (H) family Prevotellaceae, (I) genus Rothia. (J)–(M) Causal relationships on overall lung cancer: (J) genus Rothia, (K) genus Alloprevotella, (L) Haemophilus parainfluenzae, (M) Megasphaera micronuciformis. IV = instrumental variable, MR = Mendelian Randomization, SE = standard error.

**Figure 4.**
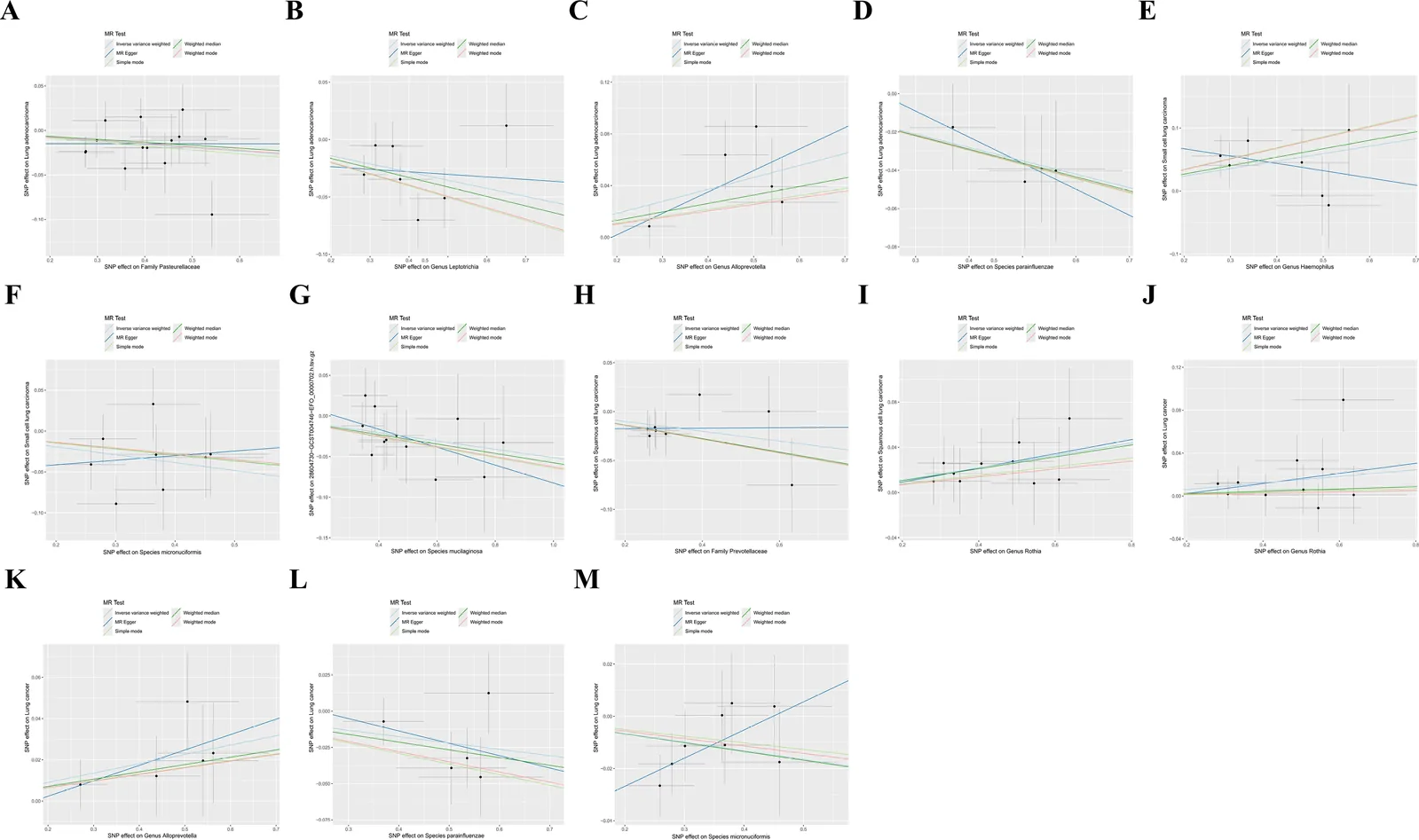
Scatter plot for the relationship between the effect sizes of SNPs on the oral microbiome (x-axis) and the corresponding effect sizes on lung cancer (y-axis). (A–D) Plots for lung adenocarcinoma: (A) family Pasteurellaceae, (B) genus Leptotrichia, (C) genus Alloprevotella, (D) Haemophilus parainfluenzae. (E)–(G) Plots for small cell lung cancer: (E) genus Haemophilus, (F) Megasphaera micronuciformis, (G) Rothia mucilaginosa. (H–I) Plots for squamous cell lung carcinoma: (H) family Prevotellaceae, (I) genus Rothia. (J–M) Plots for overall lung cancer: (J) genus Rothia, (K) genus Alloprevotella, (L) Haemophilus parainfluenzae, (M) Megasphaera micronuciformis. MR = Mendelian randomization, SNP = single nucleotide polymorphism.

**Figure 5.**
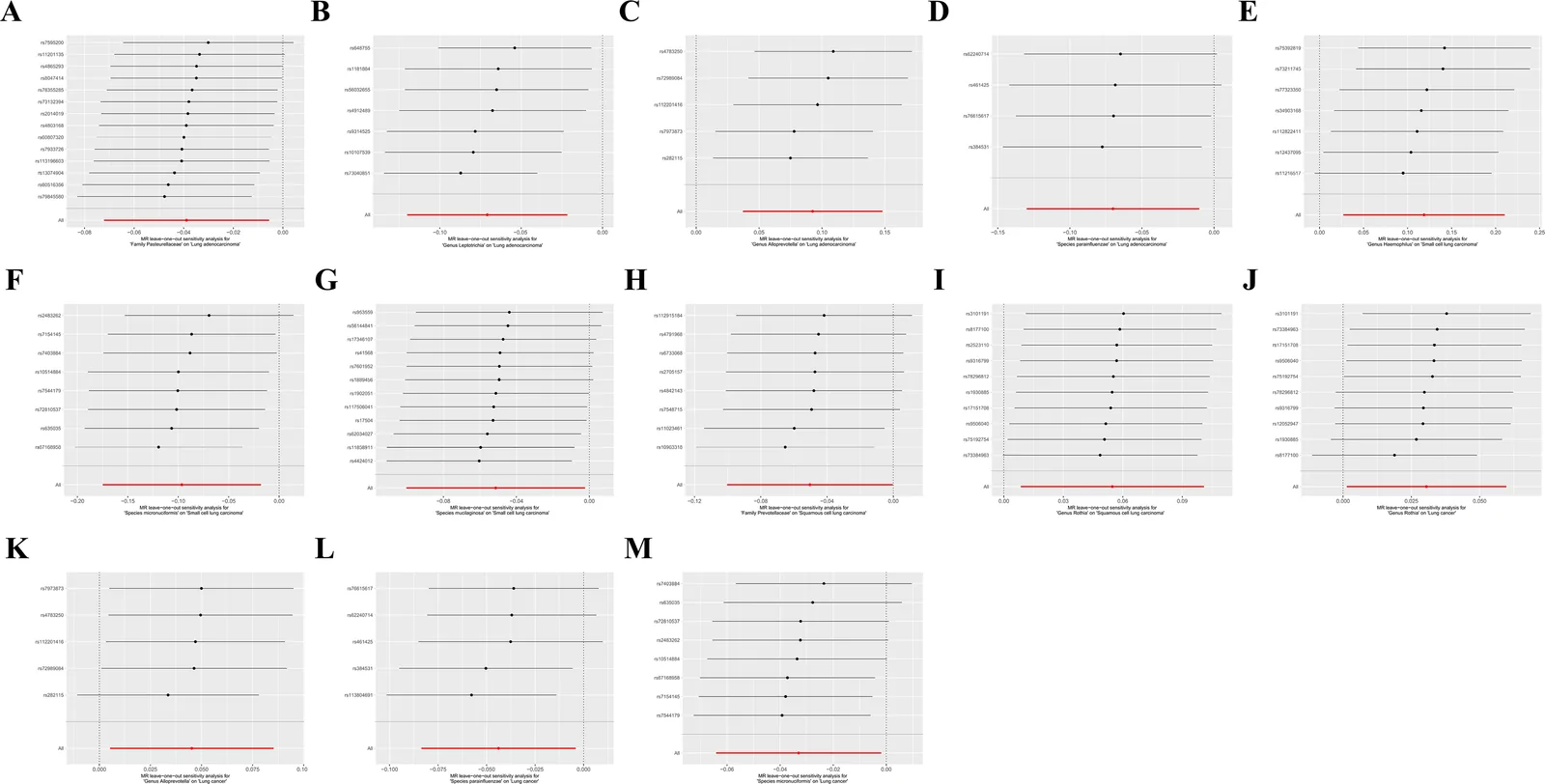
Leave-one-out plots for 13 pairs of causal relationships between oral microbiome and lung cancer. (A) ~ (D) Plots for lung adenocarcinoma: (A) family Pasteurellaceae, (B) genus Leptotrichia, (C) genus Alloprevotella, (D) Haemophilus parainfluenzae. (E) ~ (G) Plots for small cell lung cancer: (E) genus Haemophilus, (F) Megasphaera micronuciformis, (G) Rothia mucilaginosa. (H) ~ (I) Plots for squamous cell lung carcinoma: (H) family Prevotellaceae, (I) genus Rothia. (J) ~ (M) Plots for overall lung cancer: (J) genus Rothia, (K) genus Alloprevotella, (L) Haemophilus parainfluenzae, (M) Megasphaera micronuciformis. MR = Mendelian randomization.

### 3.2. Reverse MR analysis supports the directionality of the OM-LC associations

In order to exclude the effect of LC itself on the abundance of OM, we conducted reverse MR analyses using LC and its subtypes (including 4 groups of LUAD, LUSC, SCLC, and overall LC) as the exposure factors. By screening the SNPs, we obtained 6751 SNPs associated with LC (Supplementary table S5, Supplemental Digital Content 5). The F statistics of these SNPs ranged from 19.54 to 213.12, with a mean value of 30.12. The circular heatmap compared the significant differences in *P* values of the 5 analytical methods of reverse MR analysis (Fig. 6B), and the specific details of the results were partially displayed in Supplementary table S6, Supplemental Digital Content 6.

**Figure 6.**
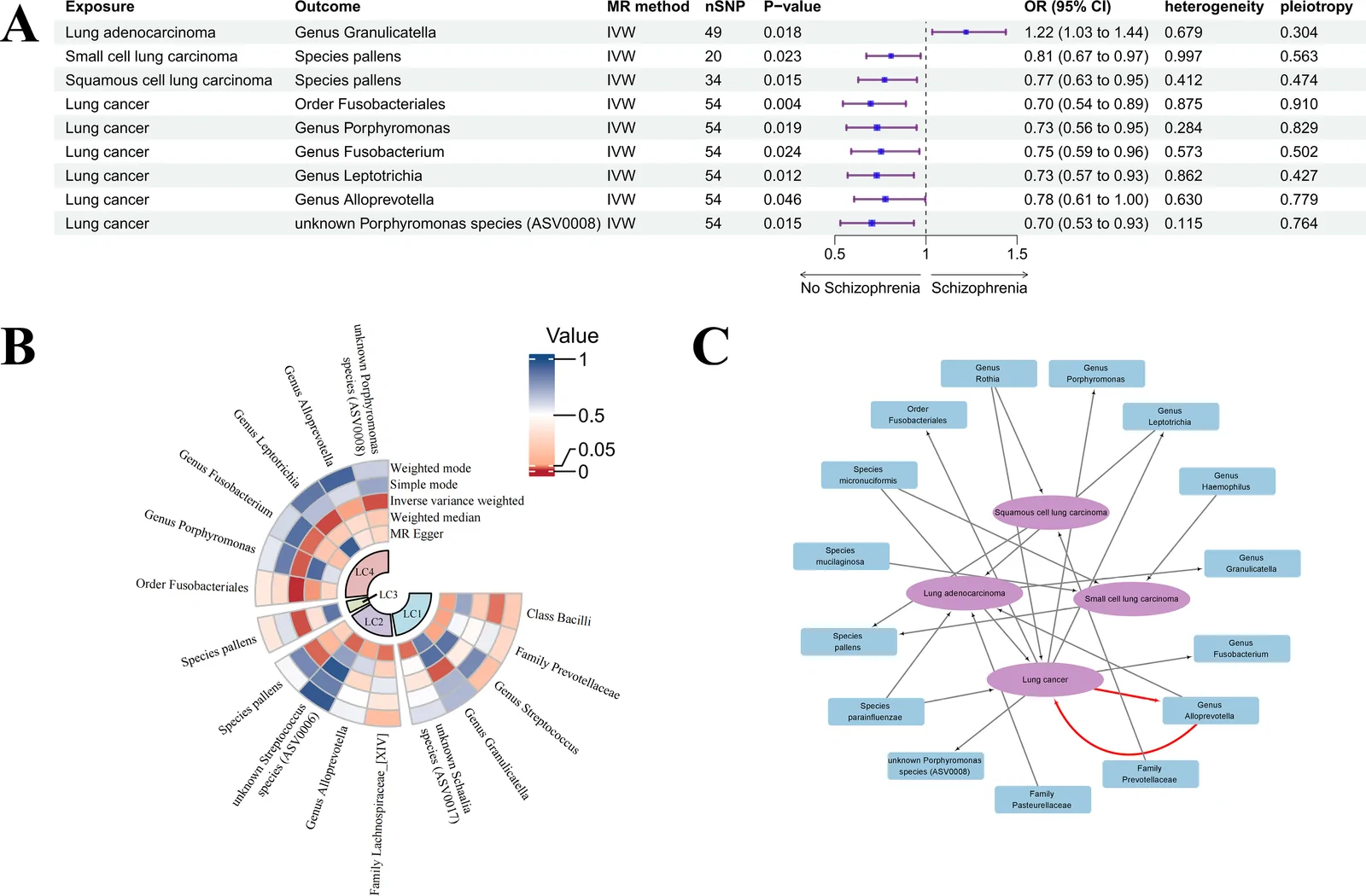
Results of reverse MR analysis of lung cancer on oral microbiome. (A) Results of the IVW model and forest plot for reverse MR analysis. (B) Circular heatmap of P values for the 5 MR analysis methods. (C) Network plot of the aggregated results of forward and reverse MR analysis. IVW = inverse variance weighted, LC1 = lung adenocarcinoma, LC2 = small cell lung cancer, LC3 = squamous cell lung carcinoma; LC4 = overall lung cancer, MR = Mendelian randomization, nSNP = number of single nucleotide polymorphism.

The results of the IVW model (Fig. 6A) indicated that LUAD could cause changes in the abundance of genus *Granulicatella* (OR = 1.22, 95%CI: 1.03^−^1.44); both SCLC and LUSC could cause changes in the abundance of *Prevotella pallens* (SCLC, OR = 0.81, 95%CI: 0.67–0.97; LUSC, OR = 0.77, 95%CI: 0.63–0.95). For overall LC, 6 OM abundances were affected, including order Fusobacteriales (OR = 0.70, 95%CI: 0.54–0.89), genus *Porphyromonas* (OR = 0.73, 95%CI: 0.56 –0.95), genus *Fusobacterium* (OR = 0.75, 95%CI: 0.59–0.96), genus *Leptotrichia* (OR = 0.73, 95%CI: 0.57–0.93), genus *Alloprevotella* (OR = 0.78, 95%CI: 0.61–1.00), and unknown *Porphyromonas* species (ASV0008) (OR = 0.70, 95%CI: 0.53–0.93). After combining the forward and reverse MR results, we found a possible interaction between genus *Alloprevotella* and overall LC (Fig. 6C). Consequently, this microbial taxon would be excluded from further research to ensure the credibility of the MR results.

In addition, both heterogeneity and horizontal multiplicity assessments (Supplementary table S7 and S8, Supplemental Digital Content 7) demonstrated the robustness of the positive results (*P* > .05). There was no discernible bias among SNPs, as seen by the funnel plots (Fig. 7). Scatter plots visualized the MR-Egger intercepts, and horizontal pleiotropy was excluded for all results (Fig. 8). Leave-one-out analysis suggested that each individual SNP had a small effect on the overall MR results (Fig. 9).

**Figure 7.**
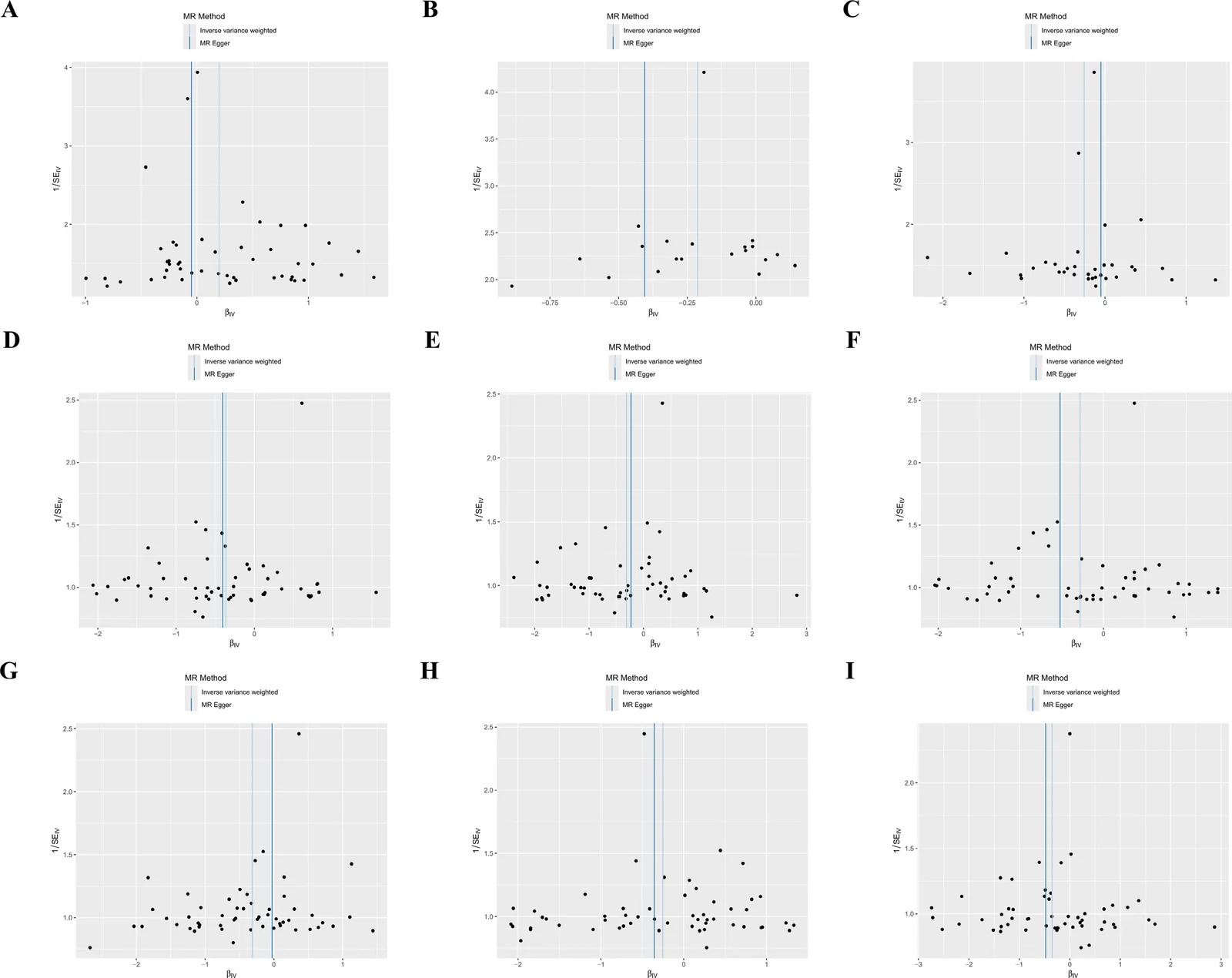
Funnel plot of 9 pairs of causal associations between lung cancer and oral microbiome. (A) SNPs extracted from lung adenocarcinoma applied to the analysis of causality with genus Granulicatella. (B) ~ (C) Extracted SNPs applied to analyze causal relationships with Prevotella pallens: (B) from small cell lung cancer, (C) from squamous cell lung carcinoma. (D) ~ (I) Extracted SNPs from lung cancer applied to the analysis of causality with oral microbiome: (D) order Fusobacteriales, (E) genus Porphyromonas, (F) genus Fusobacterium, (G) genus Leptotrichia, (H) genus Alloprevotella, (I) unknown Porphyromonas species (ASV0008). IV = instrumental variable, MR = Mendelian randomization, SE = standard error.

**Figure 8.**
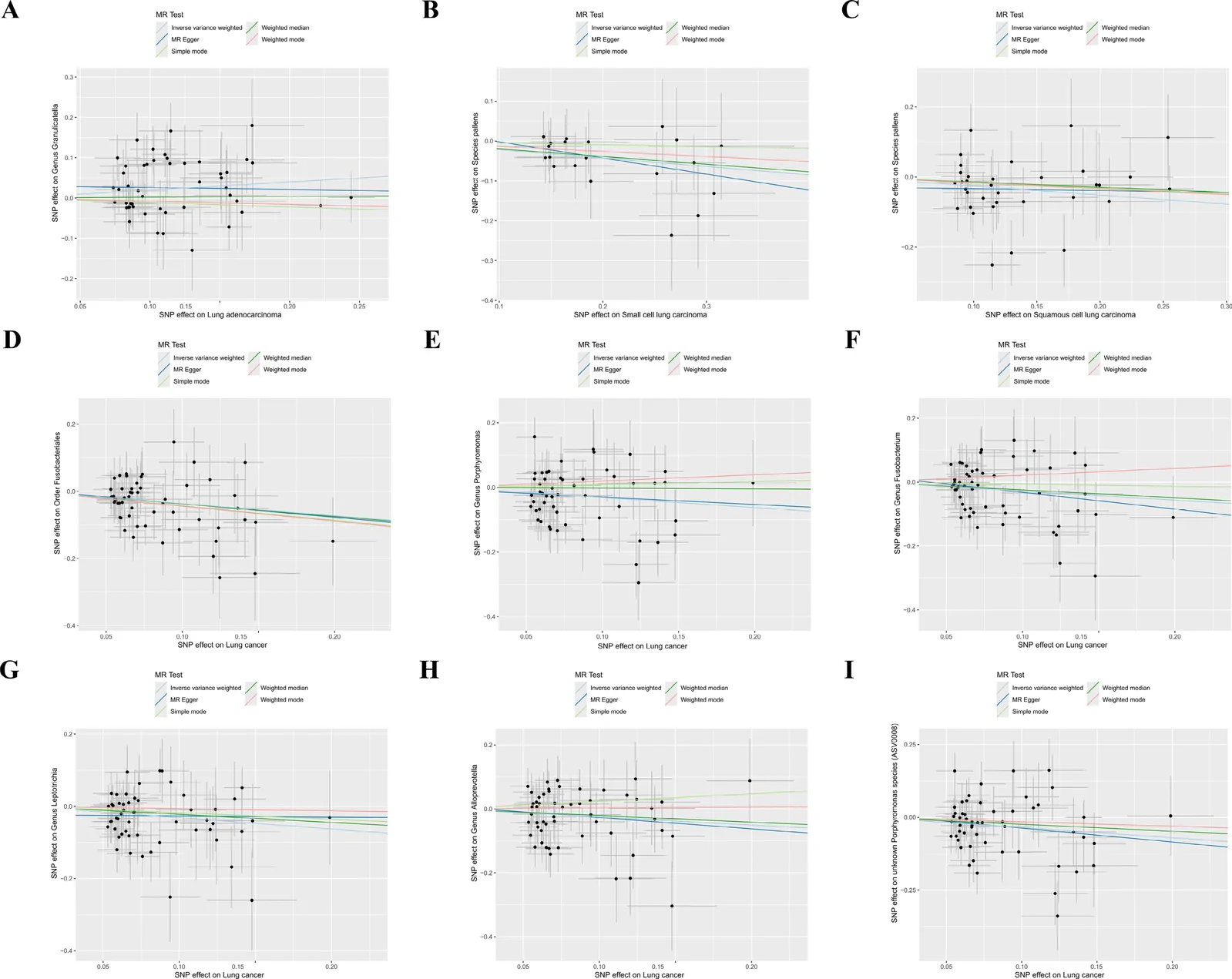
Scatter plot for the relationship between the effect size of SNPs on lung cancer (x-axis) and the corresponding effect size on the oral microbiome (y-axis). (A) Lung adenocarcinoma on genus Granulicatella. (B) Small cell lung cancer on Prevotella pallens. (C) Squamous cell lung carcinoma on Prevotella pallens. (D) Overall lung cancer on Order Fusobacteriales. (E) Overall lung cancer on genus Porphyromonas. (F) Overall lung cancer on genus Fusobacterium. (G) Overall lung cancer on genus Leptotrichia. (H) Overall lung cancer on genus Alloprevotella. (I) Overall lung cancer on unknown Porphyromonas species (ASV0008). MR = Mendelian randomization, SNP = single nucleotide polymorphism.

**Figure 9.**
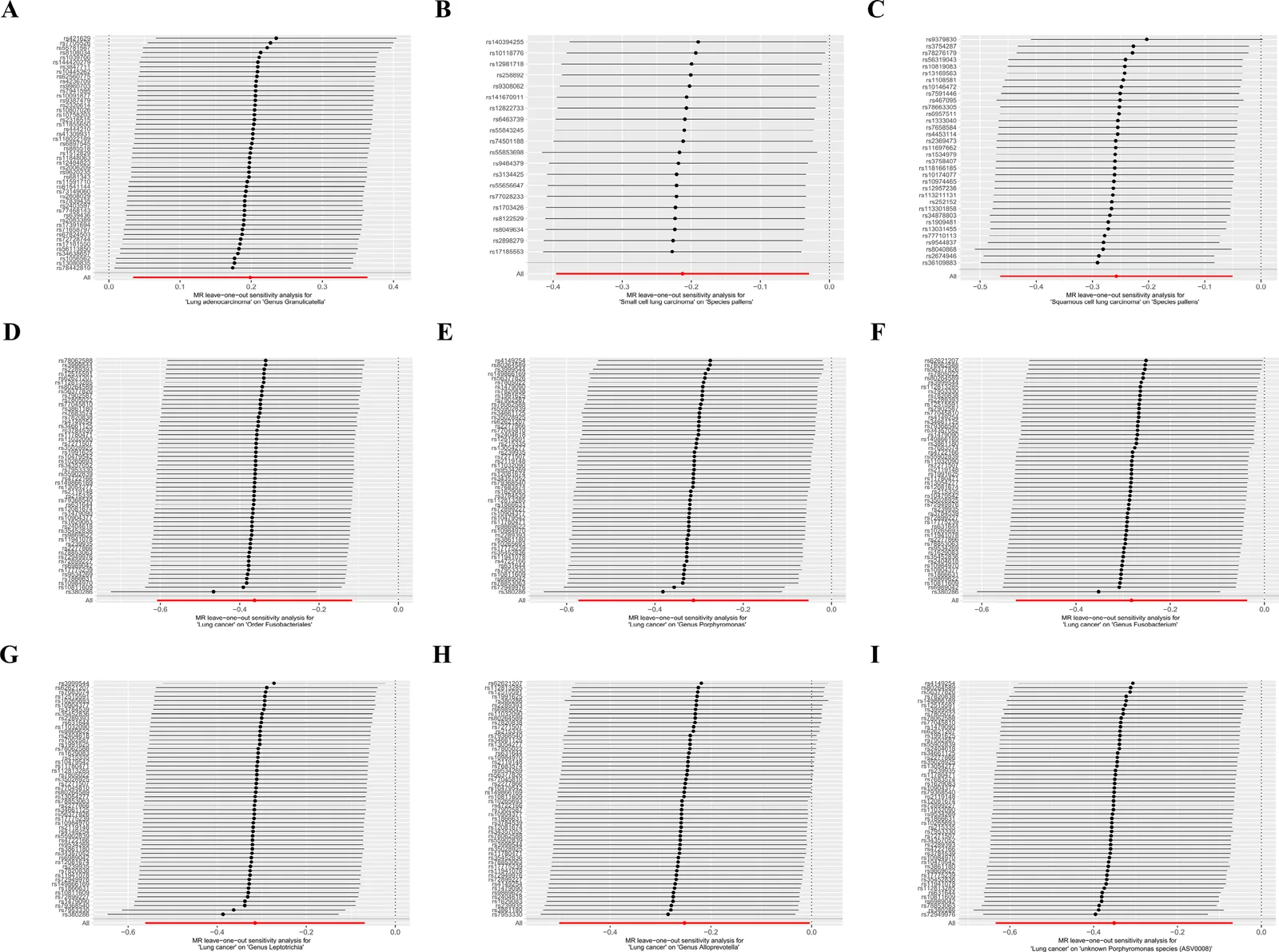
Leave-one-out plots for 9 pairs of causal pairs between lung cancer and oral microbiome. (A) Lung adenocarcinoma on genus Granulicatella. (B) Small cell lung cancer on Prevotella pallens. (C) Squamous cell lung carcinoma on Prevotella pallens. (D) Overall lung cancer on Order Fusobacteriales. (E) Overall lung cancer on genus Porphyromonas. (F) Overall lung cancer on genus Fusobacterium. (G) Overall lung cancer on genus Leptotrichia. (H) Overall lung cancer on genus Alloprevotella. (I) Overall lung cancer on unknown Porphyromonas species (ASV0008). MR = Mendelian randomization.

### 3.3. Identification of circulating inflammatory proteins associated with LC

After applying the predefined instrumental-variable selection criteria to the 91 circulating inflammatory proteins, 10,599 SNPs were retained as potential genetic instruments (Supplementary table S9, Supplemental Digital Content 8). After excluding the weak instrumental variable of *F* < 10, the *F* statistic of these SNPs ranged from 19.51 to 1911.09 with a mean value of 40.16.

A total of 49 relationships suggested that circulating inflammatory proteins may link with LC causally, which showed significance in at least one of the 5 MR methods analyses, including 11 LUAD, 13 SCLC, 13 LUSC, and 12 overall LC (Fig. 10A and Supplementary table S10, Supplemental Digital Content 9).

**Figure 10.**
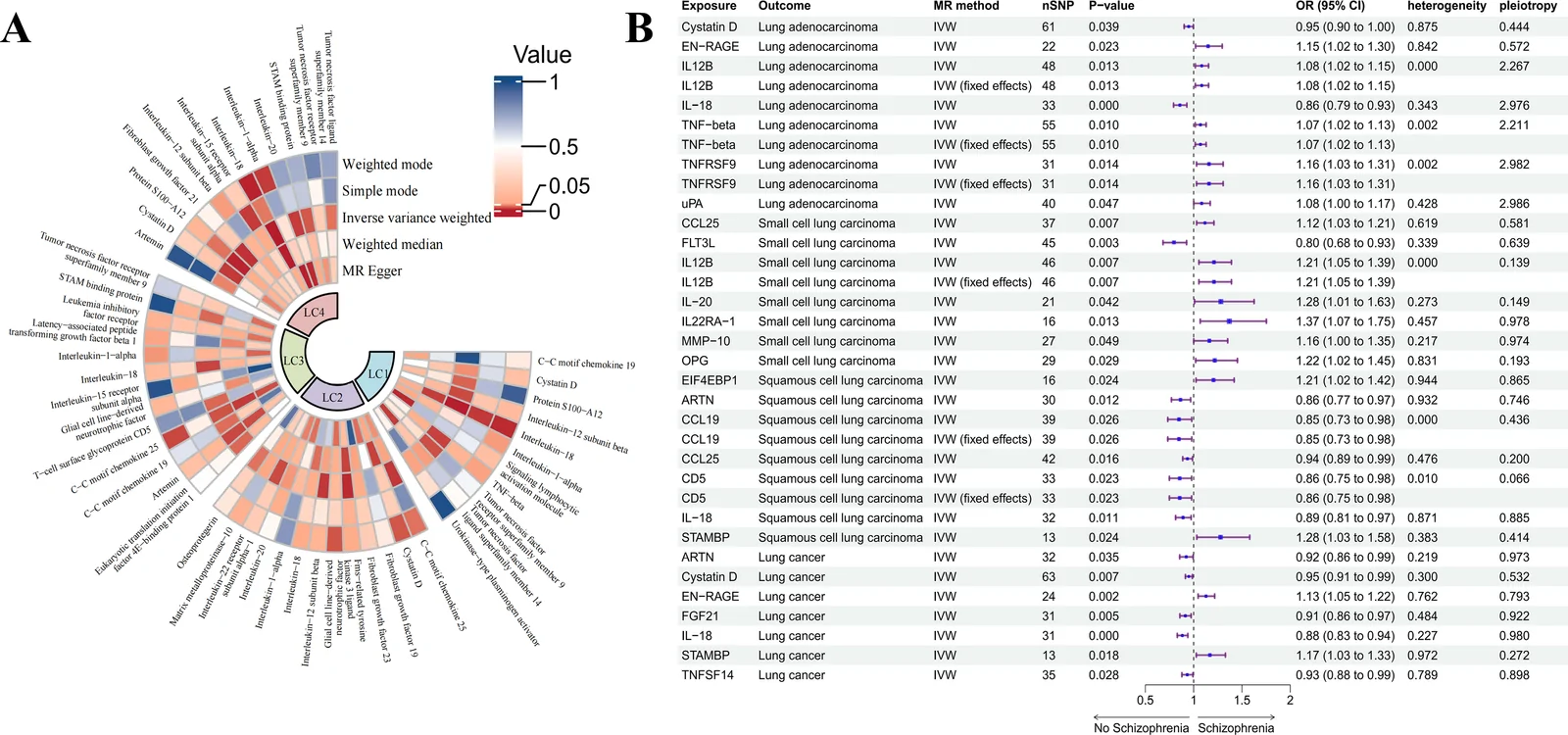
Results of MR analysis of inflammatory proteins on lung cancer. (A) Circular heatmap of P values under 5 analysis methods. (B) Results of IVW model and forest plot for MR analysis. IVW = inverse variance weighted, LC1 = lung adenocarcinoma, LC2 = small cell lung cancer; LC3 = squamous cell lung carcinoma, LC4 = overall lung cancer, MR = Mendelian randomization.

While in the IVW model, there were 30 positive results (Fig. 10B). The results of the heterogeneity test suggested that there was a potential for heterogeneity in 6 causal links, but they still showed significance after correction using the random effects model (Supplementary table S11, Supplemental Digital Content 10). However, when tested for pleiotropy, there was horizontal pleiotropy for the two causal associations between Tumor necrosis factor ligand superfamily member 14 on LUAD and Interleukin-20 on overall LC (Supplementary table S12, Supplemental Digital Content 11), and we therefore removed these 2 results.

Finally, we identified proteins associated with the risk of LUAD including cystatin D (OR = 0.95, 95%CI: 0.90–1.00), (protein S100-A12, OR = 1.15, 95%CI: 1.02–1.30), (the interleukin-12 subunit beta, OR = 1.08, 95%CI: 1.02–1.15), interleukin-18 (IL-18, OR = 0.86, 95%CI: 0.79–0.93), TNF-beta (OR = 1.07, 95%CI: 1.02–1.13), (tumor necrosis factor receptor superfamily member 9, OR = 1.16, 95%CI: 1.03–1.31), and (urokinase-type plasminogen activator, OR = 1.08, 95%CI: 1.00–1.17). Those associated with SCLC risk included C-C motif chemokine 25 (CCL25, OR = 1.12, 95%CI: 1.03–1.21), fms-related tyrosine kinase 3 ligand (FLT3L, OR = 0.80, 95%CI: 0.68–0.93), (interleukin-12 subunit beta, OR = 1.21, 95%CI: 1.05–1.39), (interleukin-20, OR = 1.28, 95%CI: 1.01–1.63), (interleukin-22 receptor subunit alpha-1, OR = 1.37, 95%CI: 1.07–1.75), (matrix metalloproteinases-10, OR = 1.16, 95%CI: 1.00– 1.35), and (osteoprotegerin, OR = 1.22, 95%CI: 1.02–1.45). Those associated with LUSC risk included eukaryotic translation initiation factor 4E-binding protein 1 (EIF4EBP1, OR = 1.21, 95%CI: 1.02–1.42), (artemin, OR = 0.86, 95%CI: 0.77–0.97), C-C motif chemokine 19 (CCL19, OR = 0.85, 95%CI: 0.73–0.98), CCL25 (OR = 0.94, 95%CI: 0.89–0.99), (T-cell surface glycoprotein CD5, OR = 0.86, 95%CI: 0.75–0.98), IL-18 (OR = 0.89, 95%CI: 0.81–0.97), and (STAM binding protein, OR = 1.28, 95%CI: 1.03–1.58). In addition, for overall LC, inflammatory proteins associated with risk included artemin (OR = 0.92, 95%CI: 0.86–0.99), cystatin D (OR = 0.95, 95%CI: 0.91–0.99), protein S100-A12 (OR = 1.13, 95%CI: 1.05–1.22), (fibroblast growth factor 21, OR = 0.91, 95%CI: 0.86–0.97), IL-18 (OR = 0.88, 95%CI: 0.83–0.94), STAM binding protein (OR = 1.17, 95%CI: 1.03–1.33), and (tumor necrosis factor ligand superfamily member 14, OR = 0.93, 95%CI: 0.88–0.99).

### 3.4. Identification of inflammatory proteins potentially linking OM to LC

Based on the above results, we obtained a total of 9 OM taxa and 20 circulating inflammatory proteins associated with the risk of LC and its subtypes. These 20 inflammatory proteins were therefore carried forward as candidate mediators and were subsequently evaluated for their potential causal relationships with the 9 selected OM taxa.

Similar to the first step of forward MR analysis, 1960 SNPs (Supplementary table S13, Supplemental Digital Content 12) associated with OM were screened and then assessed for causality with 20 inflammatory proteins. *F* statistics were in the range of 19.80 to 38.10, with a mean value of 22.08.

The causality results for the 5 MR analysis methods were shown in Supplementary table S14, Supplemental Digital Content 13, with significant differences in *P* values as shown in the circular heatmap (Fig. 11B). In contrast, only three meaningful causal links were obtained in the IVW model. Genus *Rothia* (OR = 0.97, 95%CI: 0.94–0.99) and *M. micronuciformis* (OR = 0.97, 95%CI: 0.94–1.00) had an inhibitory effect on EIF4EBP1, whereas *M. micronuciformis* (OR = 1.03, 95%CI: 1.00–1.06) had a promoting effect on Cystatin D (Fig. 11A). The results of sensitivity analyses were shown in Supplementary tables S15, S16, Supplemental Digital Content 14 and Figure 12.

**Figure 11.**
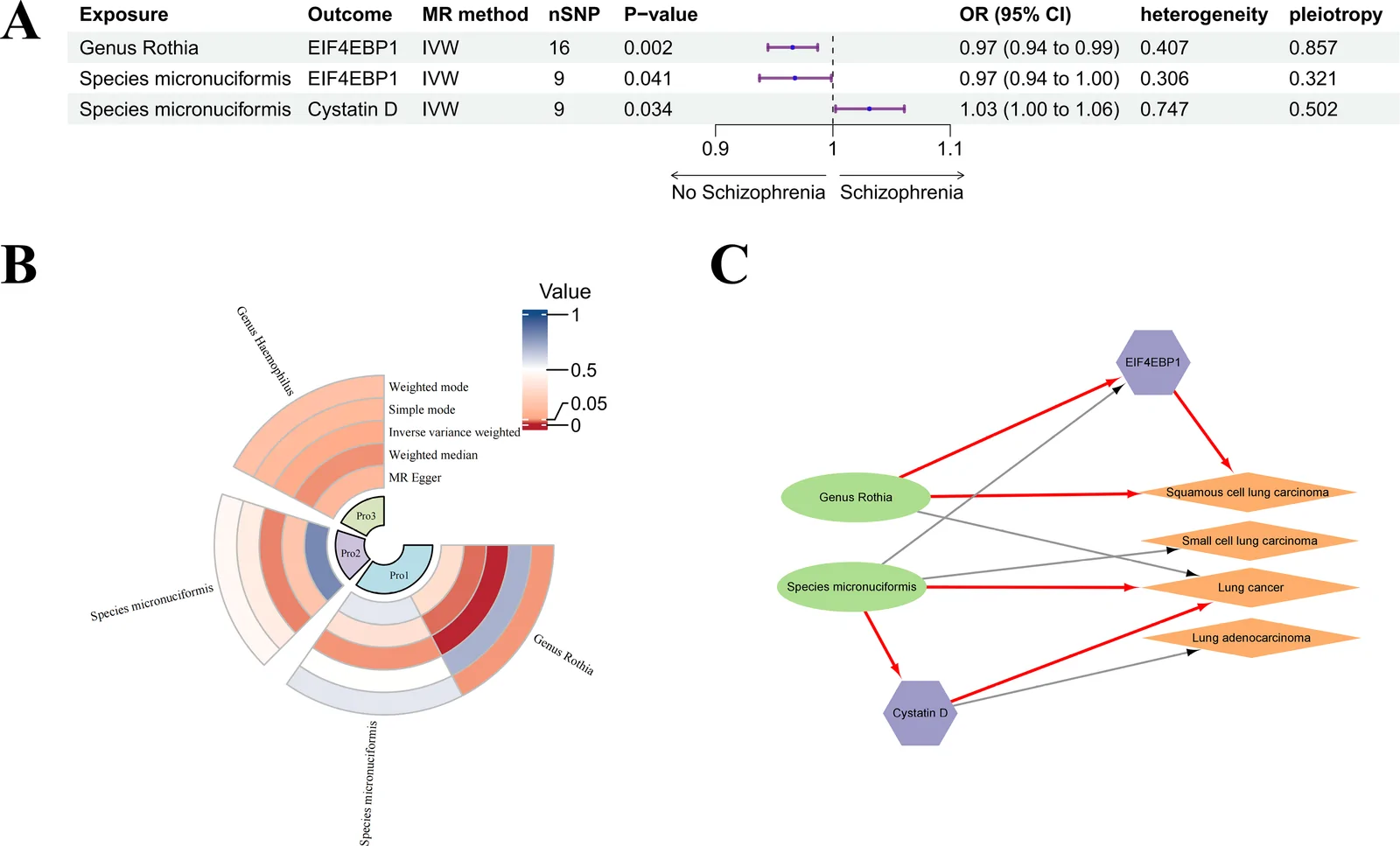
Results of MR analysis of the oral microbiome to inflammatory proteins. (A) IVW model results and forest plots of MR analysis. (B) Circular heatmap of P values under 5 analysis methods. (C) Inflammatory proteins EIF4EBP1, Cystatin D mediated network diagrams of causal relationships between genus Rothia, Megasphaera micronuciformis, and lung cancer or its subtypes. IVW = inverse variance weighted, Pro1, eukaryotic translation initiation factor 4E- binding protein 1 (EIF4EBP1); Pro2, cystatin D; Pro3, tumor necrosis factor ligand superfamily member 14 (TNFSF14), MR = Mendelian randomization.

**Figure 12.**
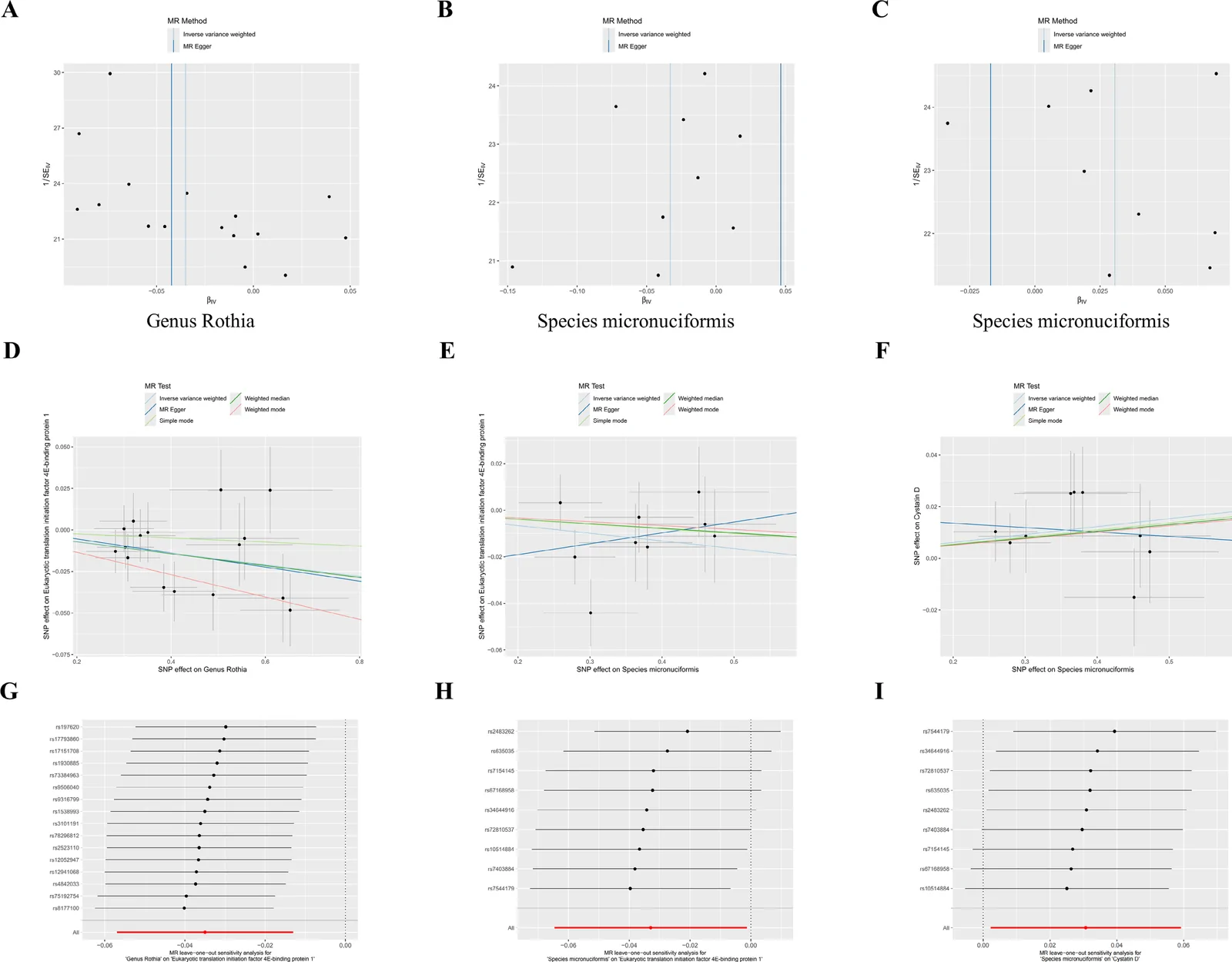
Results of sensitivity analysis of oral to inflammatory proteins. (A–C) Funnel plots of SNPs: (A) genus Rothia on EIF4EBP1, (B) Megasphaera micronuciformis on EIF4RBP1, (C) Megasphaera micronuciformis on cystatin D. (D–F) Scatter plots of SNPs: (D) extracted from genus Rothia to analysis with EIF4EBP1, (E) extracted from Megasphaera micronuciformis to analysis with EIF4EBP1, (F) extracted from Megasphaera micronuciformis to analysis with cystatin D. (G–I) Leave-one-out plots: (G) genus Rothia on EIF4EBP1, (H) Megasphaera micronuciformis on EIF4RBP1, (I) Megasphaera micronuciformis on cystatin D. MR = Mendelian randomization; EIF4EBP1, eukaryotic translation initiation factor 4E- binding protein 1.

### 3.5. Inflammatory protein-mediated causal pathways linking OM and LC

After the analysis of assess whether circulating inflammatory proteins act as intermediate mediators in the causal relationships between OM and LC, we found that EIF4EBP1, and Cystatin D were candidate mediator proteins for the causal relationships between Genus *Rothia*, *M. micronuciformis*, and overall LC (Fig. 11C).

However, in the mediation analysis, the mediation effect of genus *Rothia* on LUSC by EIF4EBP1 was in the opposite direction to the total effect. Therefore, EIF4EBP1 may not serve as a mediator of the causal relationship between Genus *Rothia* and LUSC. In contrast, cystatin D played a potential intermediate role in causality between *M. micronuciformis* and overall LC. Initially, we chose multivariate MR analysis to calculate the mediation effect, considering both *M. micronuciformis* and cystatin D as exposure factors, but there were no intersecting SNPs between 2 datasets that met the screening criteria. Therefore, we chose a two-step mediation approach for the analysis. The proportion of mediation effect was calculated to be 6.061% (Fig. 13).

**Figure 13.**
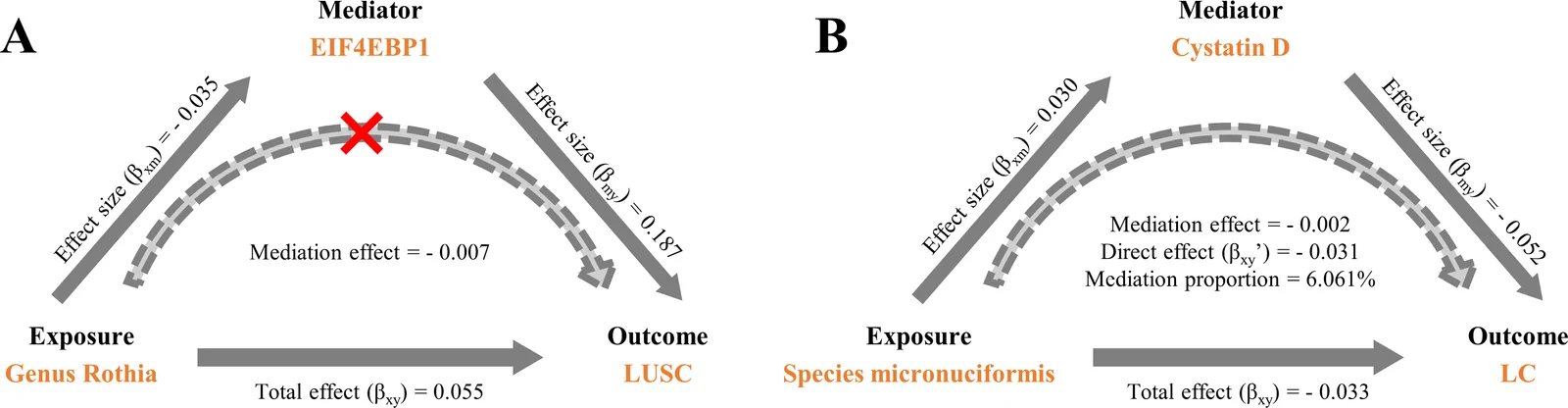
Results of mediation analysis. (A) EIF4EBP1 role in causal link between genus Rothia and LUSC; (B) Cystatin D mediated causal relationship between Megasphaera micronuciformis and overall lung cancer. EIF4EBP1 = eukaryotic translation initiation factor 4E-binding protein 1, LUSC = squamous cell lung carcinoma, LC = lung cancer.

## 4. Discussion

LC is one of the major causes of cancer-related death worldwide. Although current chemotherapy and immunosuppressive agents have improved the survival rate of patients to some extent, the incidence and mortality of LC are still high. Therefore, we urgently need to find more pathogenesis of LC from a new perspective, so as to provide enlightenment for the prevention, screening and treatment of LC in the future. In this MR study, we elucidated the causal association between OM taxa and LC risk, and then analyzed the mediating role of circulating inflammatory proteins in the causal relationship between them. Our findings indicated that 9 OM taxa and 20 inflammatory proteins were significantly linked to LC or its histopathological subtypes. Further mediation MR study results showed that cystatin D played a protective causal role between *M. micronuciformis* and overall LC. These findings provide genetic evidence supporting a potential “oral–inflammation–lung” axis in LC and identify cystatin D as a candidate intermediate factor linking *M. micronuciformis* with LC risk.

OM, as the second-largest microbial community in the human body after the gut, plays an important role in various aspects of the human immune and inflammatory systems. Recent studies have indicated that alterations in the abundance of various OM are associated with LC, yet the specific causal relationship remains unclear.^[12,13]^ According to our findings, 13 pairs of relationships showed that OM taxa were associated with the risk of LC, several of which showed consistent directions across multiple LC phenotypes (Fig. 2A). More interestingly, four of these OMs not only causally linked with the risk of overall LC but also showed significance in the causal relationship with pathological subtypes (Fig. 2C), suggesting that some oral microbial signals may extend beyond a single pathological phenotype. At the same time, the subtype-specific associations were not completely overlapping, indicating that the relationship between OM and LC may depend on the biological characteristics of different tumor subtypes. This observation is important because LUAD, LUSC, and SCLC differ substantially in their cellular origin, molecular alterations, immune microenvironment, and clinical behavior, and therefore a uniform effect of a given microbial taxon across all LC subtypes should not necessarily be expected.

We identified genus *Rothia* and genus *Alloprevotella* as risk factors associated with overall LC. In addition, these 2 bacterial genera could increase the risk of LUSC and LUAD, respectively. Genus *Rothia* is a Gram-positive bacterium that grows under aerobic or slightly aerobic conditions, which has been associated with pulmonary and systemic inflammatory conditions.^[35]^ A prospective multicenter study showed that *Rothia* in saliva samples had a better ability to predict malignant pulmonary nodules,^[36]^ while our findings extend previous observational evidence by providing genetic evidence that host genetic determinants of *Rothia* abundance may be linked to LC risk. However, genus *Rothia* has also been detected in healthy individuals and has not uniformly been associated with malignant disease. Wu et al discovered that genus *Rothia* was more abundant in bronchoalveolar lavage fluid (BALF) from normal lung segments than in ground-glass shadow lung segments when they compared the microbiological composition of BALF.^[37]^ This difference may originate from the duality of the genus, which means that the same type of bacteria may have opposite effects in different organs and microenvironments, and this may be mediated by bacterial metabolites, inflammatory proteins, cells, and other mediators.

Genus *Alloprevotella* was less well documented in the literature for its association with LC. But it was simultaneously detected as a dominant genus in the oral cavity and lower respiratory tract of patients with intermediate- or high-risk lung nodules,^[38]^ showing that its increased abundance may be harmful. Whereas, during our reverse MR analyses, we found that overall LC itself could inhibit the increase in the abundance of the genus *Alloprevotella* (Fig. 6C). Therefore, there may be a mutually restrictive relationship between *Alloprevotella* and overall LC, which requires more experiments to determine the comprehensive effect between the 2.

Our findings also indicated inhibitory effects of *H. parainfluenzae* and *M. micronuciformis* on overall LC and subtypes. Conversely, some studies found that *H. parainfluenzae* was enriched in patients with LUAD^[39]^ or was associated with poorer outcomes in NSCLC.^[40]^ However, the abundance of microbial communities is easily influenced by external factors like host resistance, immune responses, smoking, and other disease-related factors, making it hard to control for multiple variables in observational studies. Our MR analysis is based on causal inference from genetic variations, but experimental studies on the link between *H. parainfluenzae* and LC are still limited, suggesting that more data are needed to confirm the relationship in the future.

Moreover, *M. micronuciformis* is a Gram-negative coccobacillus belonging to the genus *Megasphaera*,^[41]^ which showed an inverse association with both overall LC and SCLC. Sang Hoon Lee et al compared the microbial composition of microorganisms in the BALF between 20 LC patients and 8 controls and found that the genus *Megasphaera* was more abundant in the LC patients,^[42]^ but the differences in abundance of *M. micronuciformis* were not mentioned in the text. This apparent discrepancy illustrates the distinction between genus-level observational abundance and species-level genetically predicted abundance, and different members of the same genus may have distinct or even opposing associations with LC. In general, the relationship between *M. micronuciformis* and LC is not clear, but we determined by MR analysis that it could reduce the risk of overall LC and SCLC, which is one of the highlights of our study.

Recent studies have shown that the OM can directly or indirectly affect the human immune system, promoting tumor progression through chronic inflammation or by impairing immune surveillance functions.^[43,44]^ Furthermore, chronic inflammation is widely recognized as an important component of LC, and our MR analysis further suggested that multiple circulating inflammatory proteins may be associated with LC susceptibility. Therefore, in order to determine whether circulating inflammatory proteins could provide a potential biological link between OM and LC, subsequent analyses focused on proteins that showed potential associations with both oral microbial traits and LC, allowing the identification of candidate molecules within a potential OM–inflammation–LC axis.

To address this question, we subsequently examined the relationships between the LC-associated inflammatory proteins and the oral microbial taxa identified in our study. This sequential analysis enabled us to move beyond the independent associations of individual proteins with LC and to identify candidate proteins that may connect oral microbial variation with cancer susceptibility. The results showed that EIF4EBP1 and cystatin D could both simultaneously establish a causal link with OM and LC (Fig. 11A).

EIF4EBP1 is a downstream effector of the PI3K/AKT/mTOR signaling pathway and plays an important role in the regulation of protein translation, cellular growth, and metabolic activity. Existing studies have shown that EIF4EBP1 is abnormally upregulated in LC tissues^[45–47]^ and is associated with a poor prognosis.^[48]^ This biological effect aligns with our research findings. Our study revealed that EIF4EBP1 positively correlates with LUSC and overall LC risk. However, we ultimately concluded that EIF4EBP1 cannot serve as a mediating factor in this study. Although it has a causal relationship with both genus *Rothia* and LUSC, further research revealed that the indirect effect formed by genus *Rothia* through EIF4EBP1 and LUSC is opposite to the overall effect formed by genus *Rothia* and LUSC (Fig. 13).

Cystatin D, another critical protective factor of this study, acted as an intermediate mediator in the negative relationship of *M. micronuciformis* on overall LC. Cystatin D is a novel cysteine protease inhibitor, encoded by the *CST5* gene, which is found mainly in tears and saliva and can also be detected in serum and renal tubular cells.^[49–51]^ It is inextricably linked to vitamin D, which has long been demonstrated in colon cancer. The active vitamin D metabolite 1α,25-dihydroxyvitamin D3 (1α,25(OH)_2_ D_3_) promotes cystatin D expression by inducing the binding and activation of the vitamin D receptor to the *CST5* promoter. On the 1 hand, cystatin D antagonized the Wnt/β-catenin signaling pathway and inhibited the expression of c-MYC; on the other hand, it inhibited the expression of epithelial-mesenchymal transition inducers while inducing the expression of adhesion proteins, which inhibited the growth and migration of colon cancer cells.^[52,53]^ Sabine Hünten et al also found that activation of the p53 and vitamin D_3_ pathways by combined treatment may induce stronger antitumor effects of cystatin D.^[54]^ However, in a recent 2-sample MR study, it was found that after excluding the outliers, the association between cystatin D and LUAD became significant.^[55]^ The researchers also clarified that this MR result, which may contradict existing research, requires further verification through cohort studies. In conclusion, cystatin D may have a high capacity to remove harmful factors in the human body, although the relationship between it and LC is unclear. Furthermore, this anticancer effect may be caused by exerting its role as a mediator in the relationship between *M. micronuciformis* and LC.

There were notable strengths in this study. Firstly, to the best of our knowledge, this is the first study to evaluate the causal association between OM, inflammatory proteins, and LC, which is different from previous correlation analyses. Secondly, our identification of cystatin D as a mediator in the protective effect of *M. micronuciformis* on overall LC represents a novel insight into the “oral - lung” immune axis. Besides, the relationships between *M. micronuciformis* and cystatin D and LC were not yet clear. But our research suggested their protective function, which provided new ideas for screening, prevention, and immunotherapy of LC. Furthermore, the GWAS datasets we used were all from European ancestry, which greatly reduced the impact of population bias on the results.

Nevertheless, our study also had some limitations. Firstly, in order to reduce population bias, we chose a single population of European origin, and the general applicability of our conclusions is somewhat limited. Secondly, due to the limited sample size of the OM, we used minimum criteria for screening IVs. Moreover, the absence of common SNPs during mediation analyses prevented the simultaneous inclusion of OM and inflammatory proteins as exposure factors and thus analyzing their effect sizes on LC. Furthermore, although the LC dataset was categorized into smokers’ LC and never-smokers’ LC based on smoking status, other confounding factors may still interfere with the final results. Finally, MR analyses were based on the assumptions of the theory of genetic variation, and the results of our analyses require further experimental and clinical validation studies.

In summary, this study has established a genetic association between OM, inflammatory proteins, and LC. Rather than indicating a single universal microbial cause of LC, the results suggest a heterogeneous oral–lung relationship involving multiple microbial and inflammatory components. Among these associations, the potential *M. micronuciformis*–cystatin D–LC pathway provides a particularly testable hypothesis and extends the concept of the oral–lung axis from descriptive microbial associations toward a potential host-mediated causal pathway. Further longitudinal and experimental studies are warranted to validate these associations and determine their biological and clinical relevance.

## 5. Conclusion

During this MR analysis, we analyzed the causal relationship between OM, circulating inflammatory proteins, and LC. Besides, we also found that cystatin D mediated the causal relationship between *M. micronuciformis* and overall LC. The present study provided new ideas on the risk of LC in terms of immunity and inflammation, with implications for subsequent screening, prevention, and treatment of LC.

## Acknowledgements

We would like to express our sincere gratitude to the editors and researchers for their efforts. We were also grateful to previous researchers for making the relevant GWAS datasets public.

## Author contributions

**Conceptualization:** Songhe Ping, Meng He.

**Data curation:** Songhe Ping, Dan Li, Jian Sun, Kelong Tao, E Qin, Renqi Tang, Hongguang Shan, Han Ye.

**Formal analysis:** Songhe Ping, Dan Li.

**Methodology:** Songhe Ping, Dan Li, E Qin, Meng He.

**Software:** Songhe Ping, Jian Sun.

**Visualization:** Songhe Ping, Dan Li, Jian Sun.

**Project administration:** Jian Sun, Meng He.

**Investigation:** Kelong Tao, Renqi Tang, Hongguang Shan, Han Ye.

**Resources:** E Qin.

**Funding acquisition:** Meng He.

**Supervision:** Meng He.

**Writing – original draft:** Songhe Ping, Dan Li, Jian Sun, Kelong Tao, E Qin, Meng He.

**Writing – review & editing:** Songhe Ping, Dan Li, Jian Sun, Kelong Tao, Renqi Tang, Hongguang Shan, Han Ye, Meng He.
































